# Prenatal diagnosis of hereditary diffuse gastric cancer: a case report

**DOI:** 10.1186/s12884-023-05772-6

**Published:** 2023-07-01

**Authors:** Jun Xiao, Hui Li, Fenggui Xue, Zhifei Luo, Yanyang Pang

**Affiliations:** 1 College of Traditional Chinese Medicine, Hainan Medical University; Department of Pathology, The First Affiliated Hospital of Hainan Medical University, 570100 Haikou, China; 2 Prenatal Diagnosis Center, Hainan Maternity and Child Health Hospital, 570100 Haikou, China; 3 College of Traditional Chinese Medicine, Hainan Medical University, 570100 Haikou, China

**Keywords:** HDGC, CDH1, E-cadherin, Genetic counseling, Prenatal diagnosis, Hereditary diffuse gastric cancer

## Abstract

**Background:**

Hereditary diffuse gastric cancer(HDGC) is a kind of malignant gastric cancer that is difficult to find in the early stage. However, this late onset and incomplete penetrance hereditary cancer, and its prenatal diagnosis have rarely been reported previously.

**Case presentation:**

A 26-year-old woman was referred to genetic counseling for an ultrasonography of fetal choroid plexus cyst at 17 weeks of gestation. The ultrasonographic evaluation showed bilateral choroid plexus cysts(CPC) in the lateral ventricles, and the women showed a family history of gastric cancer and breast cancer. Trio copy number sequencing identified a pathogenic CDH1 deletion in the fetus and unaffected mother. The CDH1 deletion was found in three of the five family members tested, segregation among affected family members. The couple finally decided to terminate the pregnancy after genetic counseling by hospital geneticists due to the uncertainty of the occurrence of HDGC in the future.

**Conclusions:**

In prenatal diagnosis, a family history of cancer should be widely concerned, and prenatal diagnosis of hereditary tumors requires extensive cooperation between the prenatal diagnosis structure and the pathology department.

**Supplementary Information:**

The online version contains supplementary material available at 10.1186/s12884-023-05772-6.

## Background

Hereditary diffuse gastric cancer (HDGC) is an autosomal dominant hereditary disease that occurs in 1-3% of gastric cancer cases [1, 2]. It is characterized by a malignant, poorly differentiated adenocarcinoma that can cause extensive gastric wall thickening, and the typical pathological feature is the appearance of signet ring cells [2]. The onset age of HDGC ranges from 14 to 69 years old, with an average age of 38 years old [3, 4]. The cumulative risk of developing gastric cancer for CDH1 pathogenic variant carriers in their lifetime is estimated to be 70%, and there is also a 42% risk of lobular breast cancer (LBC) for women [5, 6]. A diagnosis of HDGC is established through a proband’s pathological diagnosis and family history. According to the consensus guidelines of HDGC developed by the International Gastric Cancer Linkage Consortium (IGCLC) in 2015 [7], the clinical criteria for genetic screening of families with suspected hereditary gastric cancer include any of the following: (1) two or more individuals in a family with gastric cancer, and one of them is diagnosed with diffuse gastric cancer (DGC) before the age of 50; (2) a total of three first-degree/second-degree relatives were diagnosed with DGC, regardless of age; (3) a single case of DGC diagnosed before age 40 (the only case in the family); or (4) an individual or family history of DGC or lobular breast cancer, diagnosis age less than 50 years. Germline mutations are the cause of most confirmed HDGC cases in the CDH1 or CTNNA1 gene [8]. According to the 2020 guidelines, the clinical definition is no longer used, and the definition criteria of HDGC has been replaced by a pathogenic germline variant found in CDH1 or CTNNA1 in an isolated individual with DGC or with a family history of DGC in first-degree or second-degree relatives. Genetic testing criteria for HGDC have been updated [9]. For CDH1 pathogenic variant carriers, early detection and treatment measures of gastric cancer can be taken, and the most effective preventive measure is total gastrectomy. It is recommended to receive genetic counseling from geneticists, and a multidisciplinary team of care is needed. For women, it is recommended to conduct breast cancer screening every year. In special cases, preventive mastectomy may be considered [10].

Germline mutations can be passed on to offspring, the offspring of HDGC patients at higher risk of DGC and LBC of the females [8]. But, once the CDH1 pathogenic variant has been identified in an affected family member, prenatal testing of HDGC high-risk pregnancy is possible, and preimplantation genetic testing is also feasible [11, 12]. Requests for antenatal care for non-intelligible and treatable conditions such as HDGC are not common. Doctors and family members have different opinions on prenatal detection of later onset or reduced penetrance inherited cancer predispositions, especially considering the termination of pregnancy [13]. In our study, the CDH1 deletion variant(seq[hg19]chr16:68760000-69040000(del)) was identified in a fetus with gastric cancer and breast cancer history. We herein present the family pedigree of the fetus and the CDH1 variant found in three of the five family members tested. The results support a hereditary diffuse gastric cancer associated with this CDH1 variant. After genetic counseling and informed consent, the parents decided to terminate the pregnancy at 21 weeks of gestation.

## Case presentation

**Fig. 1:**
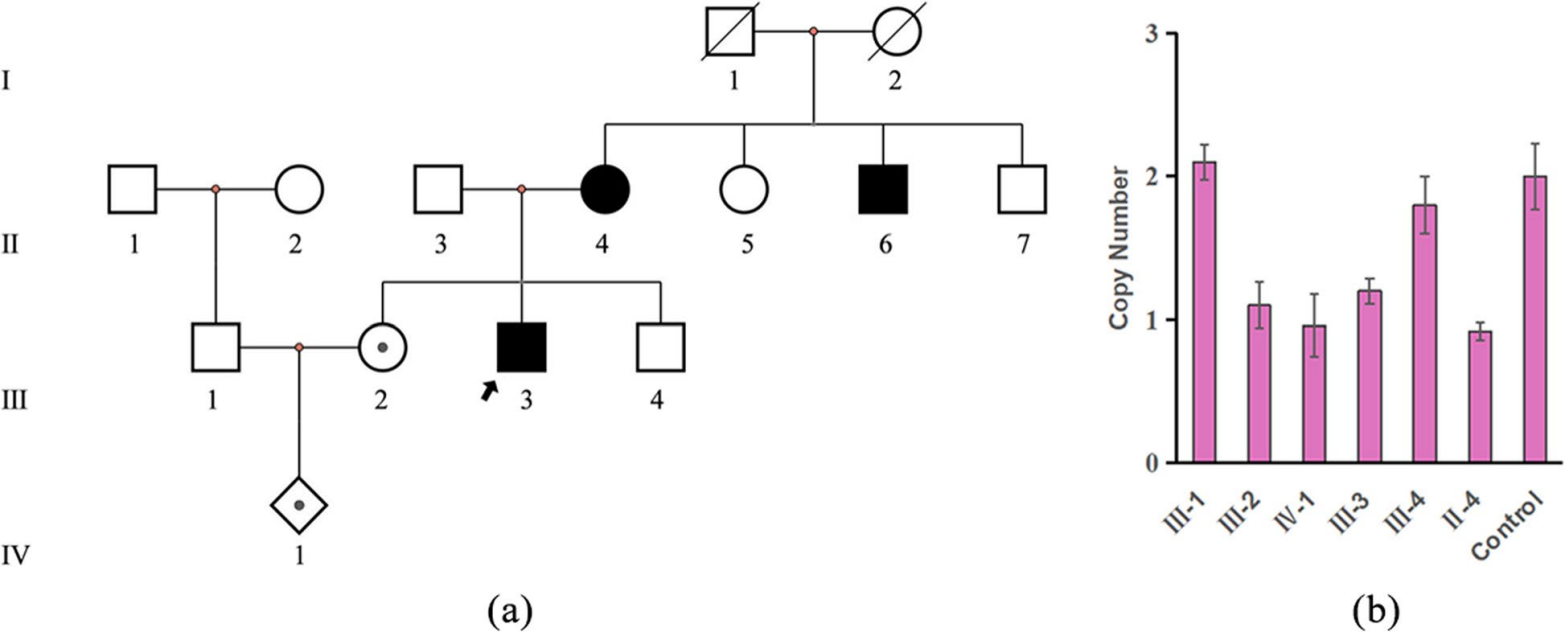
Partial pedigree of the presented family harboring a pathogenic CDH1 deletion germline variant. **a** Partial pedigree of the presented family harboring a pathogenic CDH1 germline variant. Black circles and squares indicate cancer patients; the center black dot square and prism indicate mutation carriers. **b** CDH1 copy numbers result of family members (Control: normal control)

A 26-year-old pregnant woman, gravida 2 para 0, was referred to the center of prenatal diagnosis at the Hainan Maternity and Child Health Hospital because of ultrasonography of fetal choroid plexus cyst at 17 weeks of gestation. Ultrasound showed that the biparietal diameter was 38.0mm, head circumference was 138.8mm, abdominal circumference was 122.5mm, femur length was 22.2mm, and estimated fetal weight was 189±28g; these indicate normal intrauterine growth. The ultrasonographic evaluation also showed bilateral choroid plexus cysts in the lateral ventricles, 7.9 x 5.5mm on the right and 6.9 x 4.0mm on the left (Fig. S1). The woman and her husband were healthy and non-consanguineous, with no history of infection or exposure to teratogens. Non-invasive prenatal screening (NIPS) using cell-free DNA in maternal blood at 13 weeks did not reveal any chromosome number abnormality [14]. However, the pregnant woman disclosed a familial history of gastric and breast cancer during the collection stage of the family history (Fig. 1a). There were three cancer patients in the family (Fig. 1a); the maternal uncle of the fetus (proband, III-3) was diagnosed with diffuse gastric cancer at 32 years old and underwent a subtotal gastrectomy; the mother of the proband (II-4) was diagnosed with lobular breast cancer at 56 years old; and the proband’s uncle (II-6) died of gastric cancer at 51 years old (Fig. 1a).

**Fig. 2:**
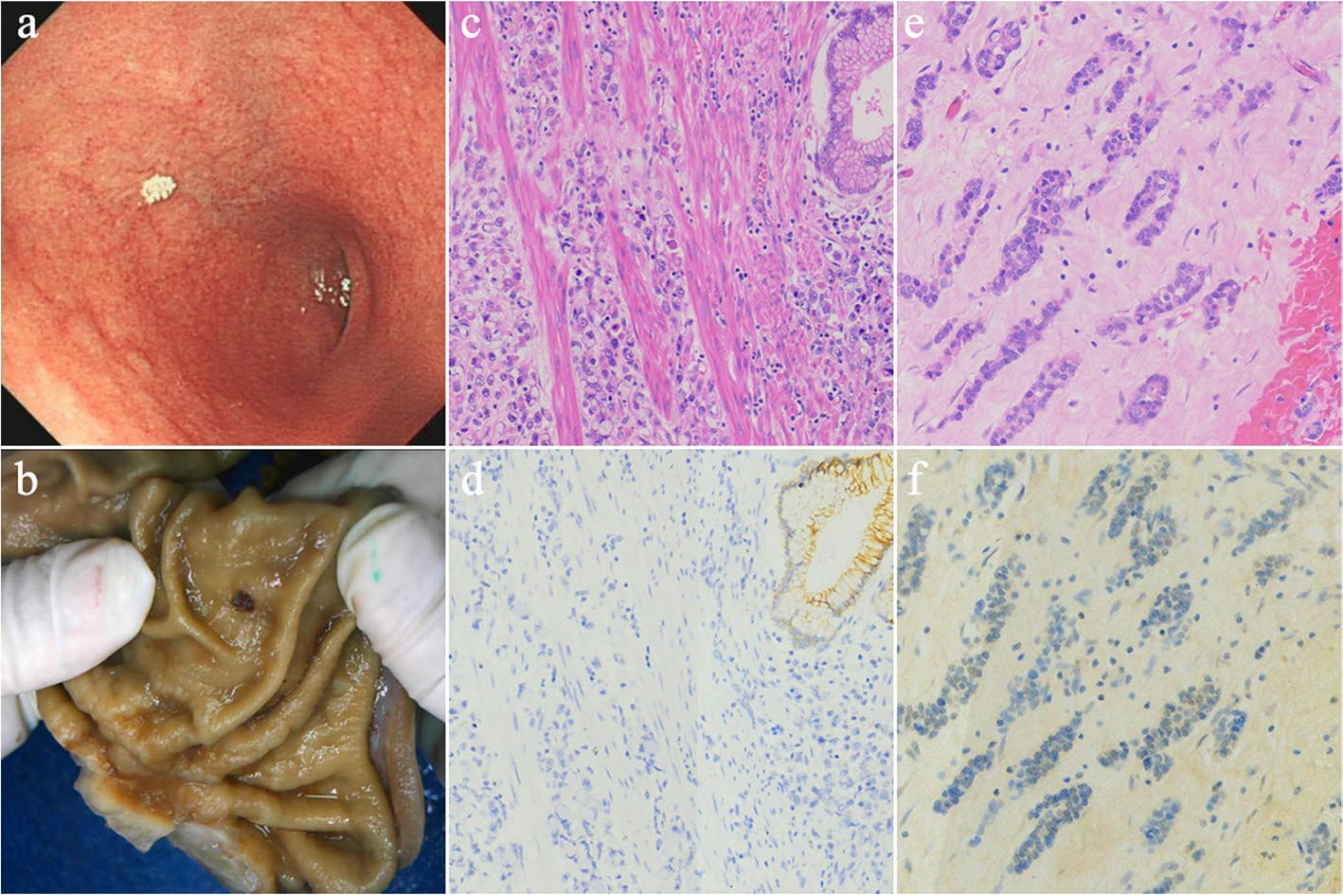
Pathological findings of patients in the family ; **a** endoscopic image of the proband’s stomach; **b** surgical specimens of gastric fixed in 10% formalin, sunken lesions were found in the lower left corner; **c** pathological picture of diffuse gastric cancer(H &E 100X), cancer cells invade the muscularis mucosa; **d** immunohistochemical staining of E-cadherin of diffuse gastric cancer (III-3, IHC 100X), positive staining is shown in brown; **e** pathological picture of invasive lobular carcinoma (ILC) (H &E, 100X); **f** immunohistochemical staining of E-cadherin of invasive lobular carcinoma (II-4, 100X). E-cadherin was expressed in the benign epithelium but was lost in the tumor(E-cadherin, 200X)

After genetic counseling, amniocentesis was performed due to a family history of cancer and an ultrasonic abnormality. Genomic structural variation analyses were conducted on amniotic fluid and peripheral blood of the couple using trio copy number variation sequencing (CNV-Seq) and quantitative fluorescent polymerase chain reaction (QF-PCR) [15, 16]. A pathogenic microdeletion of 16q22.1 (seq[hg19]chr16:(68760000-69040000)$\times$1) was identified in the fetus, inherited from the mother (pregnant woman) [17]. This microdeletion of chromosome 16 contains a known disease gene (CDH1) for HDGC. Pathological findings of two patients(II-4,III-3) in the family were collected (Fig. 2). The proband’s endoscopic and image analysis of gastric resection specimen fixed with 10% formalin revealed gastric ulcer affection (Fig. 2a, b). The proband was diagnosed with poorly differentiated gastric adenocarcinoma in clinicopathological diagnosis (World Health Organization criteria); Lauren’s criteria classified it into diffuse-type gastric cancer, signet ring cell carcinoma more than 50%. Immunohistochemical staining showed decreased E-cadherin expression in tumor cells. The proband’s mother was diagnosed with Invasive lobular carcinoma (ILC) at 56 years old and has undergone a double mastectomy. Immunohistochemical staining showed low E-cadherin expression in tumor cells. In the family of two patients with gastric cancer, one confirmed DGC, and one patient with lobular breast cancer, CDH1 gene testing was recommended according to the latest International Gastric Cancer Linkage Consortium (IGCLC) consensus guidelines [9]. Combined with fetal genetic testing results, CDH1 gene copy number detection based on quantitative real-time polymerase chain reaction(qRT-PCR) methods was performed on family members. The CDH1 deletion was found in three of the five family members tested, segregating among affected family members. However, after informing the pathogenic CNV results, the couple ultimately decided to terminate their pregnancy at 21 weeks of gestation after genetic counseling by hospital geneticists due to the uncertainty of the occurrence of HDGC in the future.

## Discussion

Pregnant women choose genetic testing due to the history of DGC and LBC as well as abnormal ultrasonography for fetal CPC. CPCs are pseudocysts found by ultrasonic examination in the fetal choroid plexus and have an occurrence rate of 1-2% in the second trimester of pregnancy [18, 19]. Generally, in the absence of genetic disorders associated with choroid plexus cysts, adverse fetal outcomes are not expected. However, recent studies have indicated certain relationships between CPCs and fetal chromosomal abnormalities, and pathogenic CNVs have been reported in the isolated choroid plexus cyst [20]. As such, invasive genetic testing is recommended in the presence of associated anomalies [20]. The results of this study did not find any chromosomal abnormalities or known pathogenic CNVs of CPCs in the fetus, and there is no reported association between fetal choroid plexus cysts and HDGC in the literature. Finally, as the fetus carried a pathogenic mutation related to HDGC, the couple chose to terminate the pregnancy.

HDGC is an autosomal dominant disorder. Studies have indicated that the majority of confirmed HDGC cases are caused by CDH1 gene germline mutations (30-50%), while CTNNA1 germline mutations are found in a small minority of HDGC cases (1.4%). Additionally, some DGC patients tested negative for CDH1/CTNNA1 gene but had pathogenic variants in related tumor susceptibility genes, such as BRCA2, ATM, SDHB, PRSS1, MSR1, STK11, and PALB2 [9]. CDH1 is a tumor suppressor gene located on chromosome 16q22.1, which encodes E-cadherin. E-cadherin is mainly expressed at the membrane of the epithelial cell, where it exerts intercellular adhesion and inhibits invasion [21, 22]. The reduction of E-cadherin is positively correlated with mesenchymal epithelial transformation (MET), a biological process for epithelial cells to acquire motility and invasion [23]. Furthermore, the reduction of E-cadherin promotes tumor cell proliferation by leading to the accumulation of beta-catenin in the cytoplasm and then translocation to the nucleus, thereby activating the Wnt/beta-catenin signaling [24]. Currently, more than 112 disease-causing mutations germline mutations (with a “DM” flag) associated with cancer or orofacial clefting phenotypes in the CDH1 gene are listed in Human Gene Mutation Database(HGMD) professional (2021.04) [25]. The type and frequency of identified CDH1 variants are as follows: 22 non-sense (19.5%), 34 frameshift (30.1%), 21 splice site (18.6%), 29 missense (25.7%), 3 start codon loss (2.7%) and 3 non-frameshift indel (2.7%). Additionally, CDH1 large deletions occur in 4% of HDGC families [26]. In addition to mutations, DNA methylation of the CDH1 promoter has been observed and may completely deactivate the gene, decreasing the expression of E-cadherin. William M. Grady et al. found that there was a second strike event in the occurrence of HDGC in the methylation study of CDH1 gene promoter in cases of hereditary diffuse gastric cancer [27]. In HDGC patients with CDH1 heterozygous germline mutations, E-cadherin is not expressed in cancer cells, and the evidence of p120 protein cytoplasm positive also supports the theory of biallelic inactivation [28].

Genetic testing based on Next Generation Sequencing (NGS) has been widely used to identify individuals with pathogenic variants in cancer susceptibility genes [29]. Prenatal genetic testing for pregnancies at increased risk is possible if the pathogenic variant in the family is known. Prenatal genetic diagnosis (PND) is mainly used for detecting abnormal fetal development and some congenital malformations, neuro developmental disorders, multiple congenital anomalies, ultrasound abnormalities, and a family history of hereditary diseases [17]. However, requests for prenatal genetic testing for conditions that do not affect intellect and have some treatment available (e.g. HDGC) are not common [30]. Neurofibroma, Retinoblastoma, Tuberous sclerosis, and some hereditary cancers with high penetrance expected at an early age are common in prenatal genetic diagnosis. Until now, PND has been reported in later onset and/or reduced penetrance inherited cancer predispositions, such as Familial adenomatous polyposis, Hereditary breast and/or ovarian cancer, Multiple endocrine neoplasia type 1, Hereditary diffuse gastric cancer, Familial medullary thyroid cancer, Lynch syndrome, Li-Fraumeni syndrome, Juvenile polyposis syndrome, MUTYH-associated polyposis, Peutz-Jeghers syndrome, and von Hippel-Lindau syndrome [30]. The American College of Medical Genetics and Genomics (ACMG) released the ACMG secondary findings gene list V3.0 used in clinical exome and genome sequencing in 2021, 38.5% (28/73) of the secondary findings genes are tumor genes, and the recommendations continue to support the reporting of known or expected pathogenic variants [31]. For HDGC, there has been no complete case study of PND; only sporadic cases have been mentioned in systematic reviews or meta-analyses [32]. For the CDH1 gene, a PND case has been reported in a chinese mother and several fetuses displayed cleft lips with palate or other facial dysmorphic features [33]. Although PND is available for hereditary cancer syndromes in some countries, the application of these techniques remains controversial in the social, ethical, and political domains [30, 32, 34]. The overall PND proportion of hereditary cancer is still very low relative to the incidence rate. High-risk individuals with HDGC have preferred preimplantation genetic diagnosis (PGD) over PND because it avoids the need for a termination of pregnancy [32]. A study investigating the desire to have children in Peutz-Jeghers syndrome (PJS) patients and breast cancer patients showed the same risk appetite for preference for PGD over PND; termination of pregnancy after PND in the case of a fetus with PJS was considered “acceptable” for 15% of the respondents, whereas 52% considered PGD acceptable [13, 35].

Thorough evaluation of the family history of hereditary cancer prior to conception is of utmost importance. Pre-pregnancy counseling can provide couples with solutions to avoid hereditary cancers during pregnancy, thus avoiding the dilemma of “suffering from the same type of pain” or “terminating the pregnancy” in prenatal diagnosis. Consequently, it is strongly recommended that patients who have been diagnosed with DGC and LBC undergo comprehensive genetic testing. Furthermore, carrier screening for individuals belonging to high-risk groups of HDGC is highly advocated. These essential measures serve as the cornerstone for the implementation of prenatal diagnosis (PND) and preimplantation genetic diagnosis (PGD). In the current case, a genetic diagnosis of HDGC is made by combining the pathological findings and the genetic testing results. The diagnosis of hereditary cancers not only depends on genetic testing but also requires the cooperation of the pathology department to achieve accurate typing of the cancers. Once the CDH1 pathogenic variant has been identified in a fetus, the doctor does not take the initiative to advise pregnant women to terminate a pregnancy but only informs the risks of HDGC. Termination of pregnancy depends more on the choice of the couple. For couples who wish to have another child, the recommendations of genetic consulting experts should be followed.

## Conclusions

The prenatal diagnosis of hereditary cancers is a relatively infrequent occurrence when juxtaposed with the developmental disorders. However, according to the incidence rate data, hereditary cancer should not be overlooked during pregnancy. This study is in addition to the clinical significance of the molecularly defined CDH1 pathogenic variant, which occurs in the DGC risk family. Our study emphasizes the importance of accurate genetic counseling and the CDH1 test analysis, particularly in patients with a family history. Furthermore, the current case is a rare report of prenatal diagnosis of HDGC, which is of great significance for the prenatal diagnosis of malignant hereditary cancers. In prenatal diagnosis, family history of cancer should be taken into account, and prenatal diagnosis of hereditary tumors requires extensive cooperation between the prenatal diagnosis structure and the pathology department.

## Supplementary Information

**Additional file 1: Figure S1.** Ultrasound Images of fetus(17 2/7 weeks of gestation). (a) Fetal skull measurement data; (b) Choroid plexus cysts(CPC) in the lateral ventricles, 0.79 × 0.55mm on the right; (c) choroid plexus cysts(CPC) in the lateral ventricles, 6.9 × 4.0mm on the left.

## Data Availability

All data analyzed during this study are included in this report. Copy Number Variant data for fetus is available in the NCBI dbVAR under the accession numbers nstd225(https://www.ncbi.nlm.nih.gov/dbvar/studies/nstd225/).

## Abbreviations

HDGC: Hereditary diffuse gastric cancer
LBC: Lobular breast cancer
DGC: Diffuse gastric cancer
PND: Prenatal diagnosis
PGD: Preimplantation genetic diagnosis
CNV-Seq: Copy number variants sequencing
QF-PCR: Quantitative fluorescent polymerase chain reaction
qRT-PCR: Quantitative real-time polymerase chain reaction
ACMG: American College of Medical Genetics and Genomics
