# Supplementary material for: Prenatal diagnosis of hereditary diffuse gastric cancer: a case report
Source: BMC Pregnancy Childbirth. 2023 Jul 1;23:488. doi: 10.1186/s12884-023-05772-6 (PMC10314645; doi:10.1186/s12884-023-05772-6)
Supplement: Supplementary file 1 — Additional file 1: Figure S1. Ultrasound Images of fetus(17 2/7 weeks of gestation). (a) Fetal skull measurement data; (b) Choroid plexus cysts(CPC) in the lateral ventricles, 0.79 × 0.55mm on the right; (c) choroid plexus cysts(CPC) in the lateral ventricles, 6.9 × 4.0mm on the left. [file 12884_2023_5772_MOESM1_ESM.pdf]

## Supplementary file

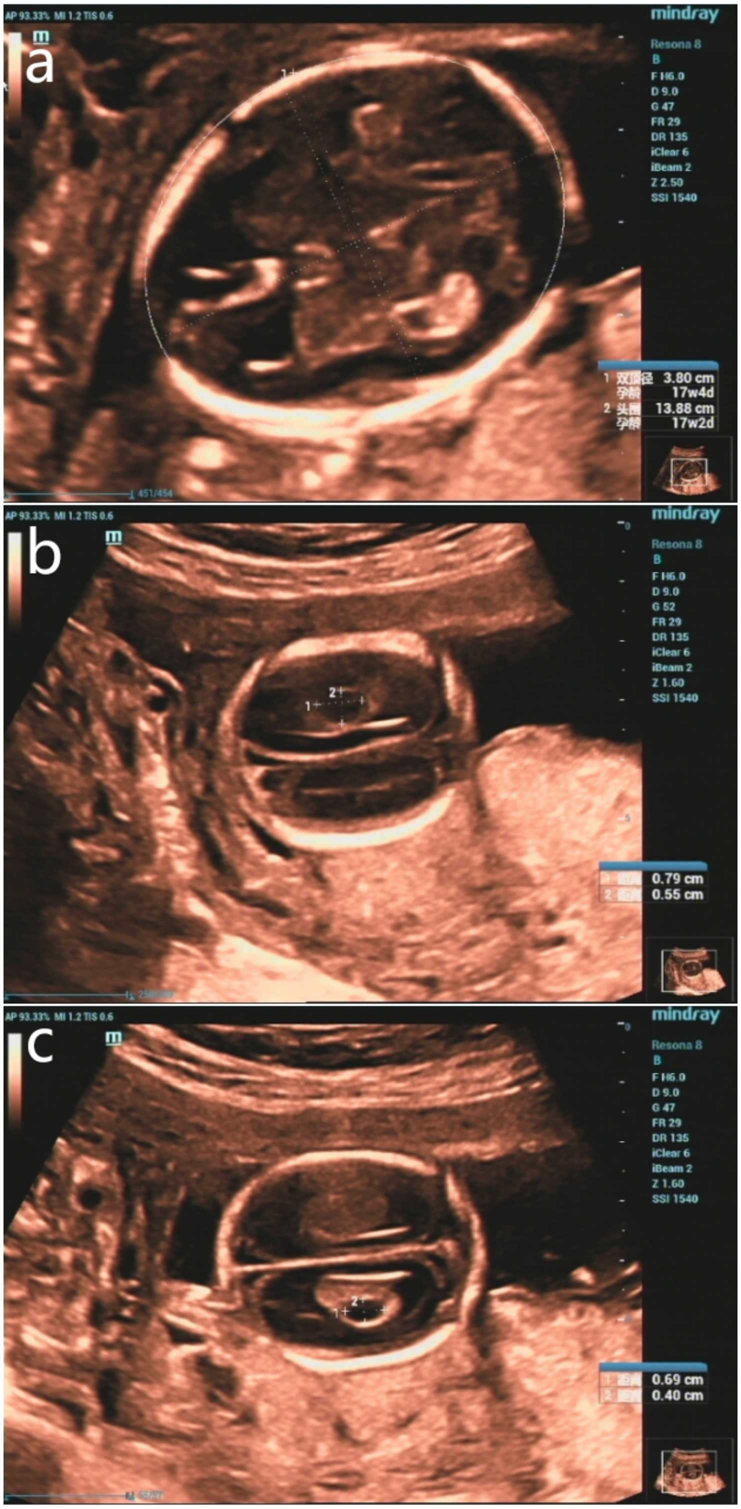




Figure S1: Ultrasound images of the fetus (17 2/7 weeks of festation). (a) fetal skull measurement data; (b) choroid plexus cysts (CPC) in the lateral ventricles, 0.79 x 0.55 mm on the right; (c) choroid plexus cysts (CPC) in the cateral ventricles, 6.9 x 4.0 mm on the left.
